# Serosurvey of *Coxiella burnetii* (Q fever) in Dromedary Camels *(Camelus dromedarius)* in Laikipia County, Kenya

**DOI:** 10.1111/zph.12337

**Published:** 2017-02-08

**Authors:** A. S. Browne, E. M. Fèvre, M. Kinnaird, D. M. Muloi, C. A. Wang, P. S. Larsen, T. O'Brien, S. L. Deem

**Affiliations:** ^1^ Molecular Epidemiology and Public Health Laboratory Hopkirk Research Institute Massey University Palmerston North NZ; ^2^ Institute of Infection and Global Health University of Liverpool Neston UK; ^3^ International Livestock Research Institute Nairobi KE; ^4^ WWF‐International The Mvuli, Nairobi KE; ^5^ Centre for Immunity, Infection and Evolution University of Edinburgh Edinburgh UK; ^6^ College of Veterinary Medicine North Carolina State University Raleigh NC USA; ^7^ Department of Epidemiology School of Public Health University of Michigan Ann Arbor MI USA; ^8^ Mpala Research Centre Nanyuki KE; ^9^ Wildlife Conservation Society Global Conservation Programs Bronx, New York NY USA; ^10^ Saint Louis Zoo Institute for Conservation Medicine Saint Louis MO USA

**Keywords:** Camels, Kenya, One Health, Q fever, Zoonoses

## Abstract

Dromedary camels *(Camelus dromedarius)* are an important protein source for people in semi‐arid and arid regions of Africa. In Kenya, camel populations have grown dramatically in the past few decades resulting in the potential for increased disease transmission between humans and camels. An estimated four million Kenyans drink unpasteurized camel milk, which poses a disease risk. We evaluated the seroprevalence of a significant zoonotic pathogen, *Coxiella burnetii* (Q fever), among 334 camels from nine herds in Laikipia County, Kenya. Serum testing revealed 18.6% positive seroprevalence of *Coxiella burnetii* (*n* = 344). Increasing camel age was positively associated with *C. burnetii* seroprevalence (OR = 5.36). Our study confirmed that camels living in Laikipia County, Kenya, have been exposed to the zoonotic pathogen, *C. burnetii*. Further research to evaluate the role of camels in disease transmission to other livestock, wildlife and humans in Kenya should be conducted.

**Impacts**

- Camels are at risk of exposure to *C. burnetti* in Laikipia County, Kenya.
- Older camels are significantly more likely to be *C. burnetii* seropositive.
- Camels are carriers of *C. burnetii* in Laikipia County, Kenya, and have the potential to be involved in the epidemiology and transmission of these pathogens to humans, other livestock and wildlife in the region.

## Introduction

Dromedary camels *(Camelus dromedarius)* are an important protein source for people in semi‐arid and arid regions of Africa. In Kenya, camel populations have increased dramatically in the past few decades with estimates of 717,500 camels in 2000 increasing to 2.9 million in 2013 (FAO 2016). During this period, camel milk production in Kenya rose from 335,000 tons to 937,000 tons, and meat production from 15,000 tons to 651 000 tons (FAO 2016). Kenyan land use for milk production has increased for camels at a time when land use for cattle ranching concurrently decreased by half (Bosire et al., 2015). Camels in Kenya are used primarily for milk production, with a shift from subsistence to market production having increased significantly in the last decade (Musinga et al., 2008; Hussein Abdi, 2010; Anderson et al., 2012; Noor et al., 2013).

Increasing drought events due to climate change have led to significant negative impacts on Kenyan livelihoods. Because camels survive better than cattle during periods of food and water scarcity, many Kenyans have switched from cattle to camels as a source of animal protein (Awuor et al., 2009). A recent study found that 71.5% of households interviewed in Isiolo County, northern Kenya, preferred camels over other livestock and cited camel endurance to climate factors as the main benefit (Kagunyu and Wanjohi, 2014).

Veterinary care and biosecurity controls for camels in Kenya lag behind those for more traditional livestock (e.g. cattle, sheep, goats) and may result in losses of productivity due to disease‐associated morbidity and mortality, as well as a potential escalation of disease transmission between camels, other livestock, wildlife and humans. Dromedary camels have been shown to harbour agents with zoonotic potential (e.g. *Coxiella burnetii*,* Brucella* spp., *Toxoplasma gondii*, rift valley fever, anthrax) and/or those that may be transmitted between camels, other livestock and wildlife (e.g. blue tongue, bovine diarrhoea virus, anthrax, *Trypanosoma evansi*) (Davies et al., 1985; Mustafa, 1987; Afzal and Sakkir, 1994; Al‐Ani et al., 1998; OIE 2010; El‐Harrak et al., 2011). Losses due to infectious disease in camels also impact the economies of local camel herders (Rich and Perry, 2011). Understanding which diseases are present in camels in Kenya is crucial for mitigating the impacts of these diseases on camel productivity and public health. For example, Kaindi (2009) estimated that 10% of Kenya's 40 million people drink unpasteurized camel milk, and since raw milk is a possible transmission route for *C. burnetii*, the consumption of unpasteurized camel milk in Kenya may pose a high public health cost in country (Cerf and Condron, 2006; Rahimi et al., 2011; Kaindi et al., 2012; Hussein et al., 2014; Osoro et al., 2015).

Vanderburg et al. (2014) found that contact with camels was associated with human Q fever across Africa. In Chad, a serosurvey of pastoralists and their livestock found that camel breeders had a nine times higher risk of being *C. burnetii* seropositive compared to the general public (Schelling et al., 2003). In Kenya, knowledge of Q fever is lacking although two studies found a 26.8% and 30.6% seroprevalence among humans tested for *C. burnetii* antibodies (Knobel et al., 2013; Mwololo et al., 2015). More recent research revealed that 16.2% of febrile patients admitted to hospitals in Northeastern Kenya were suffering from acute Q fever (Njeru et al., 2016).

A pilot study in 2012 found a high *C. burnetii* (Q fever) seroprevalence (30%) in one herd in Laikipia county, Kenya (S.L. Deem, M. Kinnaird, A.S. Browne, E.M. Fèvre, unpublished results). Other research on the same herd in 2011 revealed 46% seroprevalence in adult camels at the Mpala ranch (Depuy et al., 2014). Therefore, we focused on this pathogen in a cross‐sectional study of many camel herds in the region. The objective of our study was to determine the seroprevalence of *C. burnetii* (Q fever) in dromedary camels of Laikipia County, Kenya. Additionally, we sought to determine whether exposure to this pathogen was associated with land management systems, camel demographics or physiological abnormalities in dromedary camels.

## Materials and Methods

### Study area

**Figure 1:**
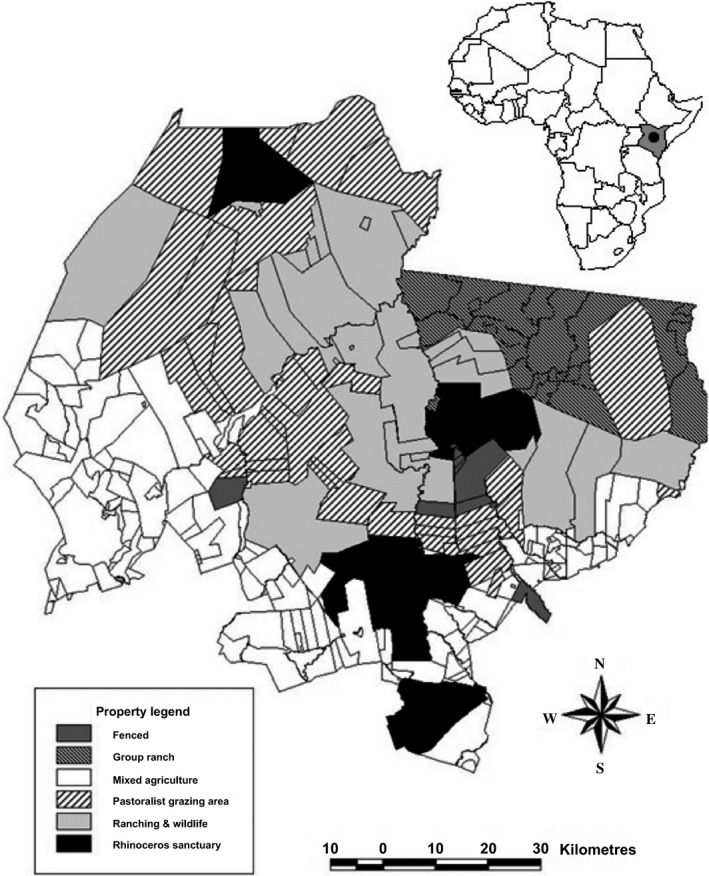
Location of Laikipia County in Kenya.

This study was conducted in Laikipia County, Kenya, a 10,000 km² area classified as semi‐arid (Fig. 1) (Pratt and Gwynne, 1977). This region is considered one of the most important wildlife areas in Kenya based on wildlife abundance and diversity (Georgiadis et al., 2007; Kinnaird and O'Brien, 2012). Properties are managed as commercial livestock ranches, pastoralist communal land and wildlife conservancies; however, most properties utilize a mixed management regime (Kinnaird and O'Brien, 2012). Camels in the region are kept for milk and meat production, as well as for transportation of supplies and people.

### Camel sampling

We sampled camels from nine Laikipia properties under different management regimes: five at predominantly commercial ranching properties (i.e. camel milk produced for sale), two at group properties (i.e. mixed commercial/pastoralist herds for camel milk production primarily for subsistence use) and two nomadic herds (i.e. camels used for the movement of supplies and people). Sampling took place from June to August 2013 and has been described elsewhere (Deem et al., 2015).

While efforts were made to sample a diverse subset of each population by selecting a range of ages and both sexes from animals distributed throughout the herd, a truly random sample was not fulfilled due to logistical constraints. Camels were marked with paint to prevent repeat sampling of individuals. Herders manually restrained camels by hobbling one front leg with a rope, so the camel could not kick or walk away, and manually restraining the head. A 4–8 ml blood sample was collected from the jugular vein using an 18‐gauge needle and placed into serum separator and EDTA vacutainer tubes.

We visually estimated tick load by examining the groin, axilla, perineum and ears. Tick load was quantified per individual into four distinct ranges: no ticks, 1–20 ticks, 21–100 ticks and >100 ticks. A veterinarian visually determined the body condition score of each camel as thin, normal or obese, using the visual appearance of the ribs and pelvis. Ages were assigned as: young (<6 months), juvenile (6 months to 2 years) and adult (>2 years) based on dental wear, physical attributes and herder/owner knowledge.

### Laboratory testing

Blood samples from the field were transported on ice to the Mpala Research Centre. Whole blood from EDTA tubes was used to determine packed cell volume (PCV) and total solids (TS). PCV was evaluated using a PCV card, while TS was determined using a refractometer; both as previously described (Deem et al., 2011).

Blood samples from serum separator tubes were allowed to clot and then centrifuged within 8 h of collection. Sera were then decanted and aliquots placed in cryotubes and stored at −20°C at the Mpala Research Centre until transported to the International Livestock Research Institute (ILRI) in Nairobi, Kenya. Sera samples were tested at ILRI for the presence of *C. burnetii* antibodies using the CHEKIT Q fever by IDEXX *C. burnetii* antibody test kit (IDEXX Europe B.V., Scoripius 60 Building F, Hoofddorp 2132 LR, The Netherlands) according to manufacturer's instructions. The CHEKIT Q fever test kit designates samples as negative, suspicious or positive, and the manufacturer reports a sensitivity and specificity of 100% (Idexx Laboratories 2011).

### Statistical analysis

We performed statistical analyses using R Version 3.2.1 (R Core Team 2015). For a seropositive result, we evaluated the following exposure variables: herd, age group, sex, body condition, PCV, TS, tick score and management. We used the ‘dplyr’ package to perform univariate analysis, with a Student's *t*‐test to test continuous variables, and Fisher's test for categorical variables (Wickham and Francois, 2015). We used the ‘lme4’ package to perform linear mixed‐effects modelling, with ‘herd’ as a random factor (Bates et al., 2014). A preliminary model was generated by stepwise backward elimination of the least significant variables, and eliminated variables were assessed for confounding. Confounding variables, determined by a change of >30% in other variable coefficients, were kept in the model even if they were non‐significant. Intraclass cluster correlation (ρ) was calculated for *C. burnetii* seropositive camels within herds using the ‘aod’ package with a Monte Carlo one‐way generalized linear mixed model (Lesnoff and Lancelot, 2012). This statistical method gives an indication of the likelihood of other animals being positive if there is one positive animal in the herd. A verbal description of the strength of correlation is as follows: 0.00–0.19: ‘very weak’; 0.20–0.39: ‘weak’; 0.40–0.59: ‘moderate’; 0.60–0.79: ‘strong’; and 0.80–1.0: ‘very strong’. Suspicious results for the CHEKIT Q fever test were counted as negative. A *P* value ≤0.05 was considered significant for all analyses.

### Ethical approval

Approval for the study was obtained from the Kenyan National Council of Science and Technology (NCST; permit number NCST/RRI/12/1/BS011/064) and the Institutional Animal and Care and Use Committee of the Saint Louis Zoo. Oral consent was obtained from camel owners.

## Results

We sampled 334 camels from nine herds (Table 1). All nine herds had at least two animals seropositive for *C. burnetii* (Table 2).

**Table 1:** Herd size, management type and proportion of camels sampled in nine camel herds in Laikipia County, Kenya

Camel herd and management type	Herd size (*n*)	Proportion of camels sampled %
Commercial 1	50	75
Commercial 2	357	10
Commercial 3	50	100
Commercial 4	131	21
Commercial 5	167	36
Group 1	76	46
Group 2	18	100
Nomadic 1	34	80
Nomadic 2	122	39
Total counts	1005	33

**Table 2:** Prevalence of *C. burnetii* in camels sampled in Laikipia County, Kenya (*n* = 334)

Herd (*n* camels sampled)	*n* positive for *C. burnetii* (%)
Commercial 1 (34)	4 (12%)
Commercial 2 (35)	4 (11%)
Commercial 3 (50)	2 (4%)
Commercial 4 (28)	8 (29%)
Commercial 5 (60)	21 (35%)
Group 1 (35)	3 (9%)
Group 2 (18)	4 (22%)
Nomadic 1 (27)	12 (44%)
Nomadic 2 (47)	4 (9%)
All herds (334)	62 (19%)

Based on an intraclass cluster correlation (ρ) of 0.11, there was noted a very weak cluster correlation. Univariate analysis of exposure variables revealed that herd, age group and TS were significantly associated with *C. burnetii* seropositivity (Table 3). Older age group and increased TS were associated with seropositivity for *C. burnetii*.

**Table 3:** *P*‐values of univariate analysis of factors related to positive *C. burnetii* (Q fever) seroprevalence among camels in Laikipia County, Kenya (*n* = 334)

Factor	*P*‐value
Herd	<0.0001^a^
Sex	0.56
Ticks	0.26
Tick score	0.28
Management	0.53
Body condition score	0.30
Pack cell volume (PCV)	0.74
Total solids (TS)	0.005^a^
Age group	0.005^a^

a Significant at *P* ≤ 0.05

The final linear mixed model for *C. burnetii* seroprevalence was generated with ‘age group’ as a significant factor (Table 4). The serostatus of Q fever among the three age groups is as follows: 7% young (*n* = 56), 14% juvenile (*n* = 81) and 24% adult (*n* = 197).

**Table 4:** Linear mixed‐effects model for *C. burnetii* (Q fever) seroprevalence among dromedary camels

Variable	Odds ratio	95% CI	Pr (>|z|)
Intercept	0.042	0.012, 0.14	0.006
Juvenile age group	2.89	0.84, 10.0	0.164
Adult age group	5.36	2.09, 21.0	0.014

Herd as a random factor variance: 0.65

## Discussion

Our study confirmed seroprevalence of *C. burnetii* among camels in Laikipia County, Kenya. Of the camels sampled, 18.6% were seropositive for *C. burnetii*. All nine herds had at least two *C. burnetii* seropositive camels. Odds of seropositivity among adult camels were 5.4 times the odds of exposure in young camels. Intraclass cluster correlation for seropositive *C. burnetii* camels was very weak among herds, indicating that the presence of a seropositive animal in a herd did not support that other camels were seropositive in that herd. This would suggest that *C. burnetii* is not highly infectious between camels in the same herd.

The ‘Commercial 5’ herd, which was sampled in both a pilot study (S.L. Deem, M. Kinnaird, A.S. Browne, E.M. Fèvre, unpublished results), and another published study (Depuy et al., 2014), revealed little change in seroprevalence over three years. This herd had the second highest seroprevalence of the nine camel herds sampled, but exposure was detected in all nine herds. Previous research in Kenya established seroprevalence of Q fever among dogs, cattle, sheep, goats and camels (Knobel et al., 2013; Depuy et al., 2014). In Saudi Arabia, seroprevalence among camels was found to be as high as 51.6% (Hussein et al., 2014). Our results for camels in Laikipia County, Kenya are similar to studies from Iran where seroprevalence ranged from 10.7% to 28.7% (Doosti et al., 2014; Pirouz et al., 2015).

In our study, positive *C. burnetii* seroprevalence significantly increased with camel age; a finding consistent with studies from Saudi Arabia and Iran (Hussein et al., 2014; Pirouz et al., 2015), as well as a previous study in Laikipia County, Kenya (Depuy et al., 2014). Camels can shed and transmit *C. burnetii* during parturition, and therefore we would have expected a higher prevalence among young and juvenile camels if transmission at birth were a significant pathway of infection. All camels in this study, even those near parturition, were kept in small enclosures (bomas) at night to avoid predation; therefore, all animals in the herd have close contact with birth fluids. If fluids associated with parturition were a significant transmission route for Q fever within camel herds, we would expect a uniform seroprevalence across the entire herd. Instead, our results suggest that this mode of transmission is low and that camels are more likely exposed as they age. One possible explanation for this increased prevalence with age could be due to tick exposure in the environment. Tick infestation was not significantly associated with positive seroprevalence in our study, but this may be due to the small sample size or the non‐probabilistic sampling.

A recent study in Kenya testing ticks removed from dogs found 50% of the ticks were positive for *C. burnetii* using real‐time polymerase chain reaction (RT‐PCR) testing (Knobel et al., 2013). In a 2012 study of of the ‘Commercial 5’ camel herd in Laikipia County, Kenya, we tested ticks removed from *C. burnetii* seropositive camels and found high concentrations of *C. burnetii* via RT‐PCR (S.L. Deem, M. Kinnaird, A.S. Browne, E.M. Fèvre, unpublished results). While tick prophylaxis was used by all herd owners in our study, applied at a minimum frequency of monthly intervals, we still found over 56% (188/334) of camels had ticks present on their bodies at the time of sampling. Camels in Laikipia County, Kenya travel across the landscape daily to browse, which may increase their exposure to ticks.

Previous research on one camel herd that was sampled for this study, ‘Commercial 5’, found that camels travelled an average of 2.2 km a day (O'Connor et al., 2015). Camel bomas or enclosures are typically relocated every few months to allow for new foraging areas to be accessed, which further increases tick exposure for camels while browsing in new environments. Acaricide use on cattle has been found to reduce the population of adult and nymphal host seeking ticks that feed on cattle and wildlife; therefore, continued acaricide use in camels could reduce Q fever transmission to wildlife in the area (Keesing et al., 2013). The presence of *C. burnetii* seropositive camels in Laikipia County, Kenya, suggests that camels may play a role in the *C. burnetii* livestock reservoir, tick vector, wildlife cycle in the area.

One limitation of our study is that we evaluated antibodies to *C. burnetii* and not antigens. Therefore, these data support a high level of exposure in the camels in Laikipia County, Kenya, but we cannot state the prevalence of infection within these herds.

Contact with camels and the consumption of unpasteurized camel milk may pose a public health risk in Kenya. We strongly support the use of heat treatment for camel milk to prevent transmission of *C. burnetii* and other zoonotic pathogens from camels to humans. Efforts should be made on a local, regional and national level to educate consumers and camel owners about mitigating the risk of this pathogen. Recent research in Northeastern Kenya revealed that 16.2% of febrile patients admitted to remote hospitals suffered from acute Q fever infection, but Q fever was not suspected by any of the treating physicians and 99.5% of the febrile patients had no knowledge of Q fever (Njeru et al., 2016).

Studies of infectious diseases of camels in Kenya are needed to identify the links that camel health may have on the health of other domestic livestock, wildlife species and human health. We recommend that studies on camel pathogens should occur alongside those that look at the incidence of these infections in humans that work with camels and/or consume camel products. The recent discovery of MERS‐CoV in Kenyan dromedary camels has helped to highlight the zoonotic potential this growing industry poses to the people of Kenya (Corman et al., 2014; Deem et al., 2015). It is imperative that scientific research, veterinary medical care and public policy for camel production be advanced in Kenya to help mitigate public health risks and disease transmission to sympatric wildlife and livestock, while advancing camel productivity in the region.
